# Industrial robots and worker health in China: evidence on sectoral heterogeneity and institutional moderation

**DOI:** 10.3389/fpubh.2026.1846138

**Published:** 2026-06-16

**Authors:** Wubin Yuan, Yuan Wang

**Affiliations:** School of Marxism, Jiangxi Normal University, Nanchang, China

**Keywords:** industrial robots, sectoral heterogeneity, the hukou system, uneven health consequences, workers’ health

## Abstract

Against the backdrop of a rising organic composition of capital driven by industrial automation, this paper examines how industrial robot adoption is associated with worker health in China and how these effects vary across groups, with particular attention to the role of labor-market institutions. Using data from the China Family Panel Studies matched with regional measures of industrial robot penetration, the analysis considers three health-related outcomes: subjective health change, objective health, and mental health. We further test the mechanisms underlying the direct health effects in manufacturing and explore the channels consistent with the cross-sector spillover patterns observed in non-manufacturing by focusing on workers’ labor-market position and on the substitutability and complementarity of labor across sectors. The results indicate that, in terms of direct effects, robot adoption is associated with significant declines in all three health measures among workers in the manufacturing sector. For workers in non-manufacturing sectors, the estimates provide suggestive evidence of cross-sector spillovers, with effects differing across health dimensions. Moreover, the health consequences of robot adoption exhibit substantial heterogeneity across worker groups, suggesting uneven health effects among workers. Overall, the findings suggest that, as capital deepening reshapes labor processes, strengthening health-risk protection and improving access to medical insurance may help mitigate adverse health consequences, especially for more vulnerable workers.

## 1 Introduction

Technological change, while advancing human society, can also exert profound effects on workers’ health and the distribution of health outcomes, with far-reaching economic and social consequences. Existing research suggests that globalization and technological progress are the two major forces behind the deepening of health inequality in advanced economies over the past four decades, with technological progress playing a particularly pivotal role (1). In particular, the rapid diffusion of industrial robots is widely viewed as an important driver of widening health disparities among workers and of rising overall inequality (2–4). Today, a new wave of technological revolution—centered on robotics—is accelerating. While boosting productivity, it is reshaping task content and work intensity, thereby exerting significant influences on workers’ health protections and on uneven health consequences.

According to the International Federation of Robotics (IFR), China has become the world leader in the operational stock of industrial robots; in 2024, installations in China accounted for 54% of global industrial robot installations (IFR, World Robotics 2025). In this paper, “robots” refer to industrial robots unless otherwise specified. The rapid diffusion of industrial robots is therefore likely to exert persistent and far-reaching impacts on both the level of workers’ health and the distribution of health outcomes. A growing literature has examined how robot adoption affects health protections and health disparities in advanced economies such as Europe and the United States (5–8), yet the findings remain mixed. This inconsistency suggests that the health consequences of technological progress for labor markets may be highly contingent on a country’s stage of development, policy environment, and institutional arrangements. Accordingly, a systematic assessment of the health effects of robot adoption in China should be grounded in China’s distinctive institutional context.

**Figure 1:**
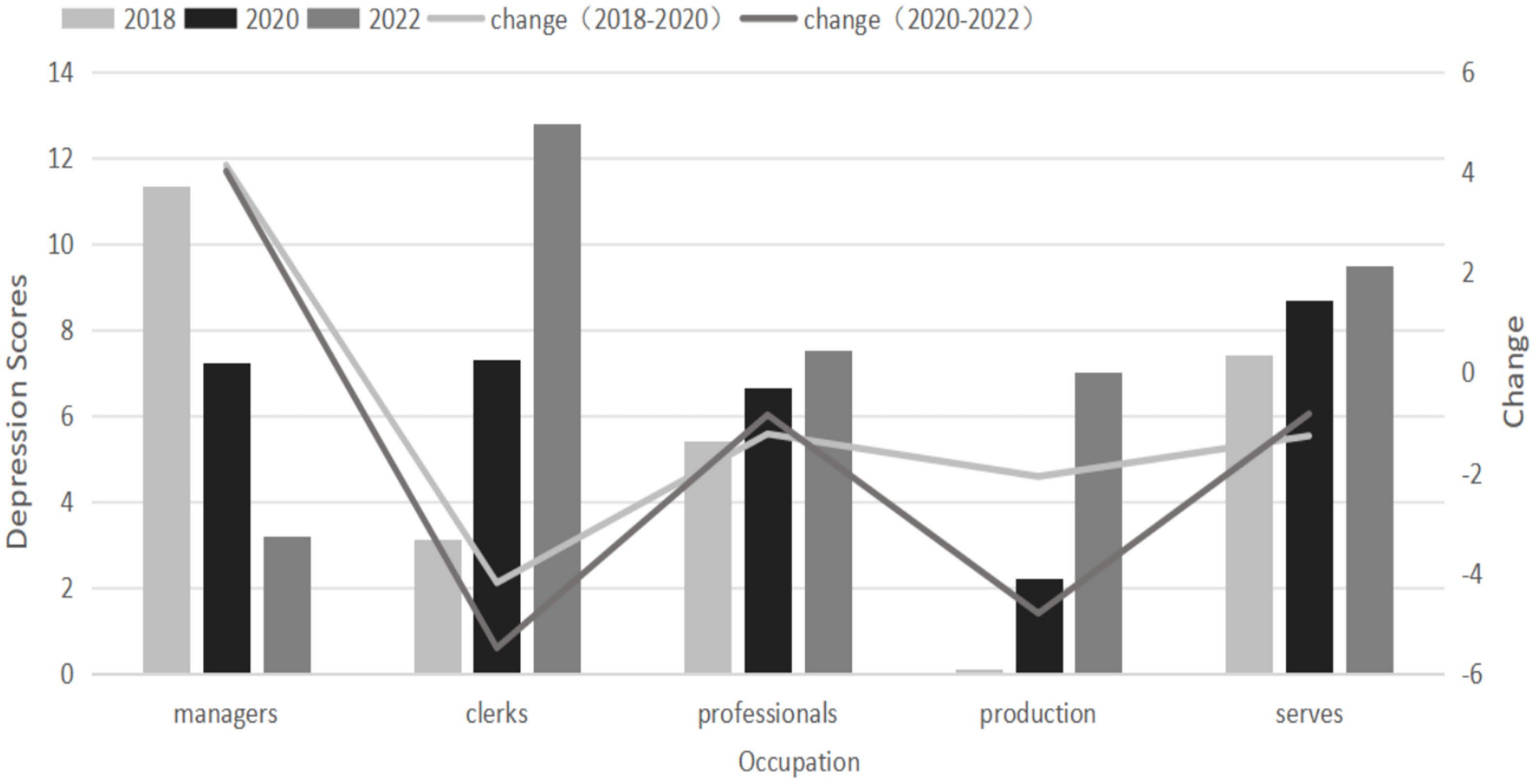
Changes in depression scores by occupation in China, 2018–2022. Data are from the National Mental Health Survey conducted by the Institute of Psychology, Chinese Academy of Sciences.

A salient empirical pattern is that, from 2018 to 2022, depression scores rose markedly faster among workers in production occupations in China than among workers in other occupations (Figure 1). Given that industrial robots are deployed primarily in production settings, workers in these occupations face a higher exposure to automation and displacement risks. This raises a central question: does industrial robot adoption amplify the deterioration of health—particularly mental health—among production workers? In addition, China’s hukou system continues to shape access to social protection during the country’s structural transformation. Under hukou-based constraints, individuals with agricultural versus non-agricultural registration differ substantially in social welfare entitlements, social insurance coverage, and access to public services. These institutional asymmetries may imply heterogeneous health effects of robot adoption across hukou groups, such that the health consequences of robot diffusion systematically diverge between agricultural-hukou and non-agricultural-hukou workers.

Although a growing body of research has documented the labor-market consequences of industrial robot adoption—showing effects on employment, wages, productivity, and sectoral adjustment across countries (9–11)—the evidence on workers’ health remains much more limited. In the Chinese context, existing studies have mainly examined labor-market adjustment to robot exposure or the drivers of robot adoption, showing that substitution effects on labor demand in manufacturing have already emerged and that some occupations face substantial risks of displacement by robots (12–16). By contrast, the literature that directly studies robots, worker safety, and well-being has focused primarily on workplace safety, occupational injuries, or broader well-being outcomes, mostly outside the Chinese institutional setting (17, 18). Consequently, systematic empirical evidence on the heterogeneity of robots’ health consequences for Chinese workers remains scarce, especially in analyses that explicitly account for China’s distinctive labor-market institutions. In addition, little is known about whether robot adoption is associated with health outcomes in non-manufacturing sectors, or through which channels such cross-sector spillovers may operate.

To provide a more comprehensive understanding of how industrial robot adoption affects workers’ health in China, this paper combines micro-level data from the China Family Panel Studies (CFPS) with industry-level measures of industrial robot use published by the IFR. We implement a Bartik (44) (shift-share) instrumental-variables strategy to identify the causal effects of robot adoption on three dimensions of health: subjective health change, objective health, and mental health. The results show that, in terms of direct effects, industrial robot adoption significantly worsens all three health outcomes among workers in the manufacturing sector. In contrast, the findings for workers in the non-manufacturing sector show clearly differentiated spillover patterns across health dimensions: robot adoption is associated with a crowding-out pattern in subjective health change, but with crowding-in patterns in objective health and mental health. With respect to distributional impacts, robot adoption contributes to “uneven health consequences,” manifested primarily in larger adverse health effects for workers in medium-skill occupations. We further provide empirical evidence on the mechanisms underlying the direct health impacts in manufacturing and offer suggestive evidence on the channels behind the cross-sector spillover patterns observed in non-manufacturing, and document heterogeneity by age and gender. Finally, we find that hukou-related institutional constraints amplify the negative health consequences of robot adoption, whereas health insurance coverage helps mitigate these adverse effects.

This paper makes three main contributions. First, relative to the literature on labor-market adjustment to robot exposure, we provide direct evidence on the health consequences of industrial robot adoption, using rich micro-level survey data to examine the direct health effects in manufacturing and the suggestive cross-sector spillover patterns observed in non-manufacturing. We also explore the underlying channels, thereby helping to interpret the health consequences of robot diffusion from a broader general-equilibrium perspective. Second, by leveraging China’s distinctive labor-market setting, we examine the consequences of robot adoption across sectors and across three dimensions of health—subjective health change, objective health, and mental health. This multidimensional framework provides a more comprehensive account of how automation affects worker health in China. Third, we show that these health consequences are shaped by institutional protection: hukou-related constraints amplify the adverse effects of robot adoption for more vulnerable workers, whereas medical insurance coverage helps buffer these effects. In this sense, the paper provides evidence on the role of institutions in conditioning workers’ exposure and resilience to automation-related health risks.

The remainder of this paper is organized as follows. Section 2 presents the theoretical mechanisms and research hypotheses. Section 3 describes the data, the measurement of key variables, and the empirical identification strategy. Section 4 analyzes the effects of industrial robot adoption on workers’ health. Section 5 investigates the underlying mechanisms and heterogeneity in these health effects. Section 6 further examines how hukou-related institutional constraints shape the health impacts of industrial robot adoption. The final section concludes and discusses the policy implications.

## 2 Theoretical framework and research hypotheses

To organize the analysis, we begin with two linked layers of mechanisms through which robot adoption may affect workers’ health. First, at the within-sector level, robot adoption directly affects workers’ health in manufacturing by reshaping task allocation, employment stability, occupational safety, and workers’ labor-market position. Second, at the cross-sector level, these labor-market adjustments may generate spillovers to non-manufacturing workers through labor reallocation pressures and the degree of substitutability and complementarity across sectors.

### 2.1 Direct effects of robot adoption on workers’ health

On the one hand, robot adoption can affect workers’ health by reshaping employment-related pressures and job attributes. The task-based model offers a useful analytical framework for understanding how robot adoption reshapes the labor process and affects workers’ outcomes (Autor et al., 2003). Within this framework, the direct health effects of robot adoption likewise operate through the productivity effect, displacement effect, and job-creation effect (19). In addition, robot adoption may affect workers’ health through an occupational-safety channel (17).

First, the productivity effect. On the one hand, by raising firm-level productivity and production efficiency, robot adoption can expand output and increase labor demand, particularly for non-automated tasks. When labor supply is relatively inelastic, higher labor demand tends to improve wages, working conditions, and investments in occupational safety and labor protection, thereby contributing to better worker health. On the other hand, when technology and labor are more complementary, robot adoption can expand “human–machine collaboration” positions, improve work processes and task organization, and ultimately enhance workers’ overall health. Second, the occupational-safety effect. Consistent with this view, Gihleb et al. (17) show that robot exposure can reduce workplace injuries. Industrial robots are often introduced into tasks that are dangerous, repetitive, physically demanding, or accident-prone. By replacing workers in such tasks, robots may reduce workers’ exposure to occupational hazards and thereby improve their health. This channel is particularly relevant in manufacturing industries, where workers are more likely to be exposed to physically demanding and hazardous production tasks. Third, the displacement effect. Within the task-based framework, capital and labor possess comparative advantages across different tasks. Given the marginal productivity of inputs and the elasticity of substitution, robot adoption extends the range of tasks that can be performed by capital, compressing the task space available to labor and substituting machines for workers in certain activities. As a result, job instability, adjustment pressures, and employment uncertainty increase, adversely affecting workers’ mental health and health expectations, thereby generating a pronounced displacement effect. Fourth, the job creation effect. While robots replace certain labor tasks, they also generate demand for new occupations associated with robot adoption, such as engineers, technicians, and maintenance personnel. The emergence of these new jobs provides workers with employment opportunities in which they may have comparative advantages and can improve health outcomes by reducing occupational exposure risks and alleviating work-related stress, giving rise to a distinct job creation effect.

On the other hand, in imperfect labor markets, robot adoption may also affect workers’ health by reshaping the labor-market position between employers and employees. From the employers’ perspective, because robots can substitute for workers in certain tasks, firms are able to choose between robotic capital and labor when their marginal productivity is comparable. Robot adoption thus expands firms’ outside options in labor allocation and strengthens employers’ dominant position in employment relationships. This shift in labor-market position may induce firms to prioritize cost minimization in job design, work intensity, and investments in occupational health and safety, thereby exerting adverse effects on workers’ health. From the workers’ perspective, the potential risk of job displacement by robots increases employment instability, which can generate insecurity and psychological stress and harm mental health. Moreover, when robot adoption creates limited additional labor demand, when the number of newly created jobs is insufficient, or when new positions require higher levels of skills and human capital, workers may find it difficult to transition smoothly in the short run. These adjustment costs and adaptation pressures further weaken workers’ labor-market position, making them more likely to accept unfavorable working conditions, higher work intensity, and inadequate health protection, and thus leading to persistent negative effects on their health outcomes. Based on the above analysis, we propose Hypothesis 1.

*Hypothesis 1:* When the productivity, occupational-safety, and job-creation effects of robot adoption dominate, higher industrial robot penetration may improve workers’ health by raising production efficiency, reducing workers’ exposure to dangerous and physically demanding tasks, and creating new human–machine collaboration jobs. Conversely, when the displacement effect dominates, higher industrial robot penetration expands firms’ outside options and weakens workers’ labor-market position, making workers more likely to accept unfavorable employment conditions and ultimately exerting a negative impact on their health outcomes.

### 2.2 Potential cross-sector spillovers of robot adoption on workers’ health: a general-equilibrium perspective

The above discussion is primarily conducted from a partial-equilibrium perspective. From a general-equilibrium standpoint, however, when the economy consists of multiple sectors—for analytical convenience, consider a two-sector setting with Sector 1 directly adopting robots and Sector 2 not adopting robots—technological progress in one sector not only directly affects the health of workers in that sector, but may also generate cross-sector spillovers in workers’ health through multiple channels. A key channel through which cross-sector spillovers may arise operates through the degree of substitutability and complementarity between labor across sectors.

Because labor and robots have different comparative advantages across tasks, workers directly affected by robot adoption can be broadly classified into two groups: those who are more easily substitutable by robots and those who are less easily substitutable. When robot adoption in Sector 1 replaces tasks previously performed by labor and reduces labor demand in that sector, labor is reallocated across sectors to restore equilibrium (assuming labor is perfectly mobile across sectors but faces constraints on mobility across regions). In this setting, if workers in Sector 2 are highly substitutable in terms of skills and task content with the robot-displaced workers from Sector 1, the inflow of displaced labor will generate a substantial labor supply shock in Sector 2. Holding labor demand relatively constant, this increase in labor supply can intensify job competition and work pressure in Sector 2, thereby adversely affecting the average health of workers in that sector—a crowding-out spillover pattern.

When, instead, there is strong complementarity across types of labor, robot adoption in Sector 1 can increase firms’ demand for workers who are less easily substituted by robots through productivity gains and job creation. These effects may further raise labor demand in Sector 2 for workers who are complementary to those in Sector 1 via positive intersectoral spillovers, improving working conditions and expected labor returns and, in turn, promoting better health outcomes—a crowding-in spillover pattern. More specifically, such positive spillovers operate through both demand-side and supply-side channels. On the demand side, when robot adoption in Sector 1 generates substantial productivity gains and job creation, it can stimulate expansion in related industries. Scale expansion is typically accompanied by increased employment opportunities, improved working environments, and greater investment in labor protection, thereby enhancing workers’ health in Sector 2 (20, 21). On the supply side, when sectors are linked through production networks, robot adoption in one sector may transmit technological spillovers along the value chain or alter production organization and labor processes in other sectors through changes in intermediate input structures and demand patterns, ultimately affecting work practices and workers’ health in those sectors. Based on this analysis, we propose Hypotheses 2 and 3.

*Hypothesis 2:* When cross-sector labor is highly substitutable, workers displaced by robots reallocate from manufacturing to non-manufacturing and generate a stronger labor supply shock. As a result, higher industrial robot penetration deteriorates health outcomes in the non-manufacturing sector—a crowding-out spillover pattern.

*Hypothesis 3:* When cross-sector labor is highly complementary, robot adoption in manufacturing raises labor demand and improves the employment environment in non-manufacturing through positive spillovers. Consequently, higher industrial robot penetration improves health outcomes in the non-manufacturing sector—a crowding-in spillover pattern.

### 2.3 Differential health consequences of robot adoption on workers’ health

Technological progress not only affects workers’ overall health but also exerts differentiated impacts across worker groups. These differences are closely related to the directional bias of technological change. The existing literature on the health effects of technological progress can be broadly classified into two strands:

The first strand is the skill-biased technological change hypothesis. Skill-biased technological progress is highly complementary to human capital. Compared with low-skilled workers with lower levels of education, high-skilled workers—typically those with a college education or above—are able to capture greater health benefits from technological advancement, thereby widening the gap in health returns between high- and low-skilled labor. Empirical studies show that, relative to low-skilled workers, the diffusion of computers and information technologies has significantly improved working environments and occupational health protection for high-skilled workers, generating larger health gains for more educated labor. This evidence provides a compelling explanation for the increasingly pronounced health disparities observed in labor markets (22, 23).

The second strand is the task-biased technological change hypothesis. This hypothesis posits that technological progress is more substitutive for routine, codifiable tasks, while being more complementary to non-routine and abstract tasks. Both theoretical and empirical studies suggest that task-biased technological change reduces labor demand for medium-skilled workers who are primarily engaged in routine activities. By contrast, for high-skilled workers performing non-routine, complex tasks and low-skilled workers engaged in non-routine, manual tasks, the likelihood of substitution by new technologies is relatively low. As a result, technological progress is more likely to increase labor demand and generate health benefits for these groups. Consequently, differences in task content give rise to divergent health outcomes across occupational groups, leading to a pattern of uneven health consequences.

A large body of empirical evidence indicates that robot adoption—particularly the diffusion of industrial robots—exhibits pronounced task-biased characteristics and is more strongly substitutive for medium-skilled workers engaged in routine and codifiable tasks. Consequently, unlike the “education effect” emphasized in the skill-biased technological change literature, industrial robot adoption primarily generates disparities in health outcomes across workers performing different tasks and occupations. In particular, workers in medium-skilled occupations dominated by routine tasks are likely to experience larger adverse health impacts, giving rise to a degree of “uneven health consequences.” Based on this analysis, we propose Hypothesis 4.

*Hypothesis 4:* The health effects of industrial robot adoption exhibit pronounced differentiation across occupational groups.

## 3 Data and identification strategy

### 3.1 Data sources

#### 3.1.1 Workers’ health

The individual-level data are drawn from the China Family Panel Studies (CFPS), a nationally representative longitudinal survey administered by the Institute of Social Science Survey at Peking University over 2010–2022. CFPS started with a baseline wave in 2010 and has been fielded biennially since then, collecting rich information on respondents’ demographic characteristics, employment, and health. We process the data as follows. First, because the analysis focuses on labor-market participants and to minimize confounding from retirement, we restrict the sample to non-students aged 16–64. Second, using respondents’ location identifiers, we match individuals to prefecture-level cities and merge in city-level economic controls and our key explanatory variable—regional industrial robot penetration—based on city codes. Third, to capture health comprehensively, we construct three outcome measures: subjective health change, objective health, and mental health.

Specifically, subjective health change is measured using the CFPS question: “How would you assess your health compared with 1 year ago?” We code the variable as 1 if the respondent reports no change or an improvement, and 0 if health is reported to have worsened. Prior research shows that subjective health change is a strong predictor of mortality (24, 25) and captures overall health status across physical, psychological, and social dimensions (26, 27). To mitigate concerns about subjectivity in self-reports, we further employ an objective health indicator based on the question: “Have you been diagnosed by a doctor with any chronic disease in the past 6 months?” This variable is coded as 1 if no chronic disease is reported and 0 otherwise. Mental health is constructed using a battery of CFPS questions that ask respondents about the frequency, over the past week, of experiencing various emotions or behaviors, including feeling depressed, finding everything effortful, having sleep difficulties, feeling happy, feeling lonely, enjoying life, and feeling sad. Responses are recorded on a four-point scale—“almost never” (less than 1 day), “sometimes” (1–2 days), “often” (3–4 days), and “most of the time” (5–7 days)—and coded from 1 to 4. Negative affect items are reverse-coded, and all items are averaged to form a composite mental health index, with higher values indicating better mental health.

The three health outcomes used in this paper capture different aspects of health and should be interpreted accordingly. The first outcome measures subjective health change relative to 1 year earlier, rather than a standard self-rated health level. The second outcome is based on self-reported doctor diagnosis of a chronic disease and is therefore not fully objective; in addition to underlying health status, it may also reflect differences in healthcare access, diagnosis probability, and insurance coverage. The third outcome captures mental health using a survey-based index. We therefore interpret these variables as complementary proxies for different dimensions of health rather than as directly comparable measures.

#### 3.1.2 Industrial robot data and other data sources

Industry-level data on industrial robots are obtained from the International Federation of Robotics (IFR). Following China’s National Industrial Classification (GB/T 4754–2002), we harmonize China’s two-digit manufacturing industry codes to the 2002 standard and, using the official concordance between China’s industry codes and the International Standard Industrial Classification (ISIC Rev.4), match China’s industry employment statistics to the IFR industry-level robot stock data.

In addition, the empirical analysis draws on the following sources: (1) manufacturing employment by industry from the China Industrial Statistical Yearbook; (2) city-level economic variables from the China City Statistical Yearbook and the Annual Survey of Industrial Firms (ASIF), which we use to estimate city-level manufacturing employment by industry; (3) U.S. industry employment data from the National Bureau of Economic Research (NBER); and (4) province-level data on industrial robot imports constructed from the China Customs Trade Database. We exclude observations with missing values in key variables and winsorize all continuous variables at the 1st and 99th percentiles to mitigate the influence of outliers.

### 3.2 Measurement of regional industrial robot penetration and the instrumental variable

Following Acemoglu and Restrepo (9), we construct a measure of regional industrial robot penetration for China. The construction is closely related to a Bartik (shift–share) approach (28) and captures the extent of industrial robot penetration in city *c* in year *t*, defined as in Equation 1:


$$\text{robo}t_{\mathrm{ast}}=\sum_{s}\frac{\text{emplo}y_{\mathrm{sc}t_{0}}}{\text{emplo}y_{\mathrm{gs}t_{0}}}\frac{\text{robo}t_{\mathrm{st}}}{L_{\mathrm{sc}t_{0}}}$$ (1)

Here, *s* denotes industry, *c* denotes city, *t* denotes year, and t0 denotes the base year. Given that the rapid expansion of industrial robots in China began around 2010, we follow Wang et al. (29) and choose 2005 as the base year. Using data from the 2005 National 1% Population Sample Survey, we compute each city’s pre-determined industry employment structure in order to purge the measure of potential endogeneity arising from robot adoption–induced changes in industry employment. Let robot_st_ denote the number of industrial robot installations in industry *s* in year *t*, $L_{{\mathrm{st}}_{0}}$ denote baseline employment in industry *s*, and let $\frac{\text{emplo}y_{\mathrm{sc}t_{0}}}{\text{emplo}y_{ct_{0}}}$ is defined as the share of industry s in total employment in city c in the base year. The weight captures the local manufacturing employment structure and reflects the sensitivity of a regional labor market to aggregate changes in industrial robot adoption at the industry level. For a given region, the larger the baseline employment share of robot-intensive industries, the greater the exposure of the local labor market to the diffusion of industrial robots.

We further construct an instrumental variable for China’s regional industrial robot penetration following Du et al. (30). The intuition is that industry-level robot adoption in the United States primarily captures global, industry-specific technological trends that are shared across countries—thereby ensuring instrument relevance—while being plausibly orthogonal to China-specific local factors that directly affect robot adoption or workers’ health, supporting the exclusion restriction. Using U.S. industry robot adoption to instrument for China’s regional exposure to robots therefore helps mitigate endogeneity concerns. The instrument is constructed as shown in Equation 2:


$$\bar{\text{Robo}t_{\mathrm{gt}}}=\sum_{s}\frac{\text{emplo}y_{\mathrm{sc}t_{0}}}{\text{emplo}y_{ct_{0}}}\frac{\text{robo}t_{\mathrm{st}}}{L_{\mathrm{sc}t_{0}}}$$ (2)

where robot_st_ denotes the stock of industrial robots in U.S. industry *s* in year *t*, and $L_{{\mathrm{st}}_{0}}$ denotes baseline employment in U.S. industry s in the base year (1990).

### 3.3 Distributional features and validity of China’s regional industrial robot penetration measure

We compute city-level industrial robot penetration for 285 Chinese cities over the period 2010–2022. In terms of spatial patterns, the fastest growth in robot penetration is concentrated in the Pearl River Delta, the Yangtze River Delta, and the Bohai Rim. These regions are characterized by higher levels of economic development and a strong manufacturing base, and they are at the forefront of advanced manufacturing in China. Because robot density in the automotive and electronics industries is substantially higher than in other industries, high robot penetration in some traditional automotive hubs may disproportionately reflect these sectors rather than overall manufacturing robot use. To assess the validity of our measure, we recompute city-level robot penetration after excluding the automotive and electronics industries. Overall, the spatial distribution of regional robot penetration excluding these two industries closely mirrors that obtained when they are included, suggesting that our measure captures broad-based manufacturing robot adoption rather than being driven by a small number of highly robotized industries.

**Figure 2:**
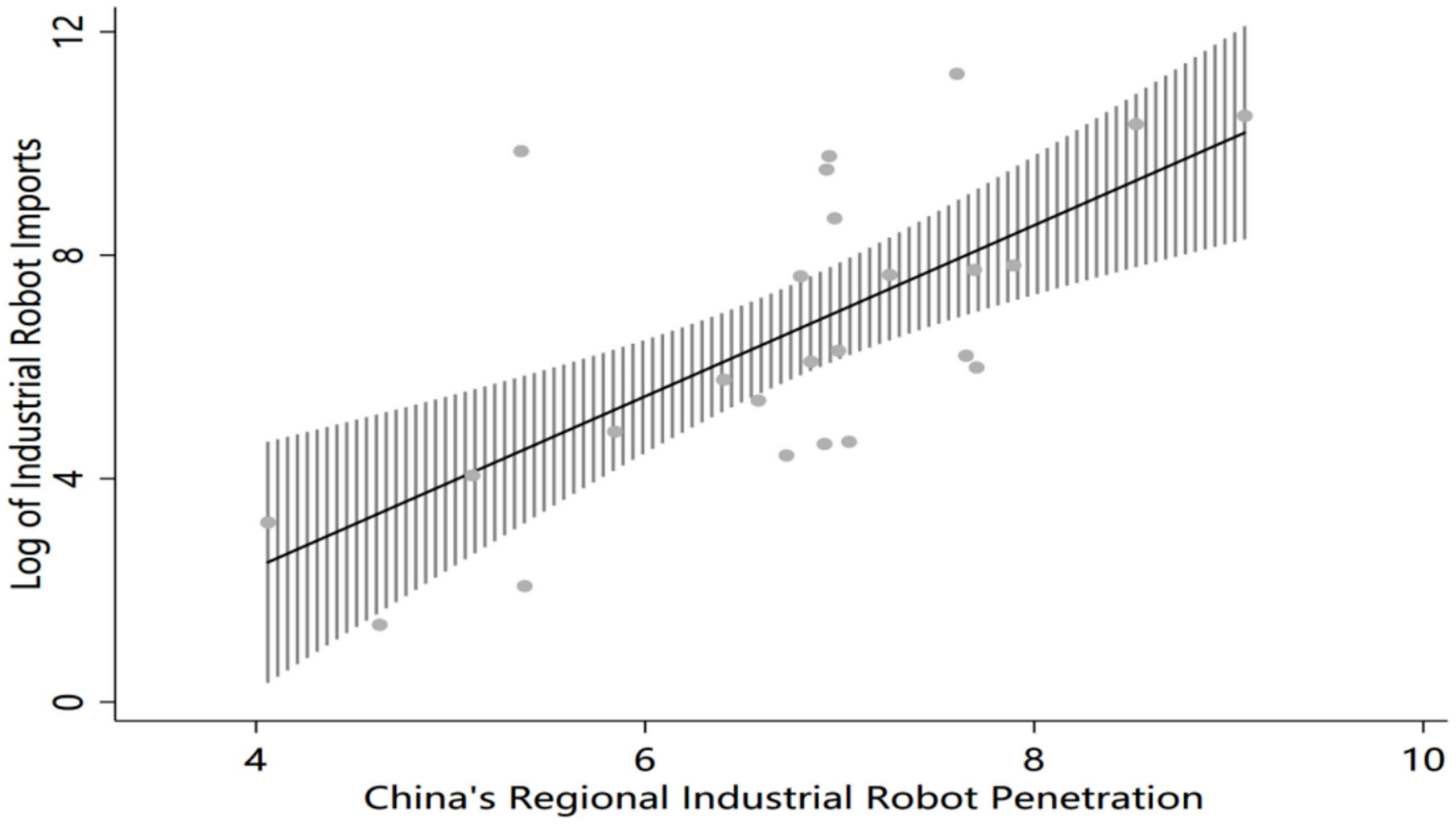
China’s regional industrial robot penetration and industrial robot imports, 2018–2022. The shaded area represents the 95% confidence interval.

According to the *Industrial Robot Industry Development White Paper* (2022), prior to 2022 the market share of domestic robot manufacturers in China remained below 35%, implying that imports were the primary channel through which domestic demand for industrial robots was met. Consequently, the number of industrial robots imported can, to some extent, proxy for a region’s actual level of robot use. We therefore aggregate our city-level robot penetration measure to the provincial level and examine its correlation with province-level industrial robot imports (Figure 2). The results show that, over 2018–2022, provinces with higher robot penetration generally exhibit larger import volumes, and most observations lie close to the fitted regression line. This pattern suggests that our robot penetration measure captures regional variation in industrial robot adoption reasonably well.

### 3.4 Research design

To examine the impact of changes in regional industrial robot penetration on individual workers’ health outcomes, we estimate the baseline regression model as specified in Equation 3:


$$y_{\mathrm{ict}}=\alpha +\beta {\text{Robot}}_{\mathrm{ct}-1}+\gamma X_{\mathrm{ict}}+\mu Z_{\mathrm{ct}-1}+\sigma_{c}+\delta_{m}+\varphi_{n}+\theta_{\mathrm{pt}}+\varepsilon_{\mathrm{ict}}$$ (3)

Here, *i* indexes individuals, *c* indexes cities, *p* indexes province, and *t* indexes years. The dependent variable y_ict_ denotes the health outcome of individual *i* residing in city *c* at time *t*. The key explanatory variable Robot_ct−1_ measures industrial robot penetration in city *c* in period *t*−1. Because the CFPS surveys are typically conducted around mid-year, whereas robot penetration is measured on an annual basis, we use lagged robot penetration to ensure temporal consistency and to mitigate simultaneity concerns. Consistent with this approach, city-level control variables Z_ct−1_ are also lagged by one period and include real GDP per capita, population density, and measures of local industrial structure calculated using both employment shares and value-added shares. The vector X_ict_ contains individual-level controls, including age, marital status (coded as 1 if married and 0 otherwise), hukou status (coded as 1 for non-agricultural hukou and 0 for agricultural hukou), educational attainment, and gender. $\sigma_{c}$ , $\delta_{m}$ , $\phi_{n}$ , and $\theta_{\mathrm{pt}}$ denote city fixed effects, occupation fixed effects, industry fixed effects, and province-by-year fixed effects, respectively. The error term is denoted by $\varepsilon_{\mathrm{ict}}$ , and standard errors are clustered at the city level. For the binary health outcomes, we use a linear probability model (LPM). Accordingly, coefficients are interpreted as changes in probability, expressed in percentage points. Descriptive statistics for the main variables are reported in Table 1.

**Table 1:** Descriptive statistics.

Variable	Variable description	Obs	Mean	Std. Dev.	Min	Max
Individual-level						
Selfhealth	Subjective health change (1 = no change or better, 0 = worse)	31,979	0.811	0.391	0	1
Objhealth	Ever diagnosed with a chronic disease by a doctor (1 = no, 0 = yes)	32,419	0.934	0.248	0	1
Menthealth	Frequency of feeling sad, upset, or similar emotions/behaviors in the past week (coded 1–4)	32,419	2.617	1.271	1	4
Age	age	32,419	51.531	12.924	16	64
Marriage	Marital status (1 = with spouse, 0 = without spouse)	32,419	0.893	0.309	0	1
Hukou	Household registration type (0 = rural hukou, 1 = urban hukou)	32,419	0.746	0.435	0	1
Education	Years of schooling	32,419	7.484	4.618	0	22
Gender	Gender (1 = male, 0 = female)	32,419	0.588	0.492	0	1
City-level						
Robot	Regional industrial robot penetration	285	4.447	3.589	0.128	12.801
lnpop	Ln(city population density)	285	6.145	0.686	4.635	7.882
lngdp	Ln(per capita GDP)	285	10.079	0.743	8.324	12.34
sec_empl	Share of secondary-industry employment in total employment (%)	285	43.156	8.53	22.224	62.824
thir_empl	Share of tertiary-industry employment in total employment (%)	285	47.134	9.911	22.477	75.083
sec_gdp	Share of secondary-industry output in GDP (%)	285	36.072	11.016	20.560	66.760
thir_gdp	Share of tertiary-industry output in GDP (%)	285	50.303	9.194	32.610	76.870

## 4 Effects of industrial robot adoption on workers’ health

### 4.1 Effects of industrial robot adoption on workers’ health in manufacturing and non-manufacturing sectors

Table 2 presents the baseline estimates of the effects of industrial robot adoption on workers’ health in manufacturing and non-manufacturing sectors. Columns (1), (3), and (5) indicate that robot adoption is associated with statistically significant deteriorations in health among manufacturing workers. Specifically, a one-unit increase in regional robot penetration is associated with a 0.59 percentage-point decline in subjective health change, a 0.15 percentage-point increase in the likelihood of having a doctor-diagnosed chronic condition, and a 0.0013-point reduction in the mental health index among manufacturing workers. For non-manufacturing workers, the estimates vary across health dimensions: a one-unit increase in regional robot penetration is associated with a 0.52 percentage-point decline in subjective health change, but with increases of 0.16 and 0.15 percentage points in objective health and mental health, respectively. To further rule out time-varying shocks specific to particular industries or occupations—such as changes in workplace safety regulation affecting certain sectors—we re-estimate the model with occupation-by-survey-year and industry-by-survey-year interaction fixed effects in Columns (2), (4), and (6). The coefficients remain stable in sign and magnitude, reinforcing the robustness of the baseline findings. Taken together, the results imply that robot adoption exerts negative direct health effects on manufacturing workers across all three outcomes. In contrast, the estimates for non-manufacturing workers provide suggestive evidence of differentiated cross-sector spillovers: subjective health change displays a negative crowding-out pattern, whereas objective health and mental health exhibit positive crowding-in patterns.

**Table 2:** Effects of industrial robot adoption on workers’ health.

Variable	Subjective health change		Objective Health		Mental Health	
	(1)	(2)	(3)	(4)	(5)	(6)
Manufacturing sector						
Robot	−0.0059*** (−2.871)	−0.0062*** (−2.952)	−0.0015*** (−2.712)	−0.0016*** (−2.685)	−0.0013*** (−2.623)	−0.0019*** (−2.773)
Controls	Yes	Yes	Yes	Yes	Yes	Yes
Fixed Effects	Yes	Yes	Yes	Yes	Yes	Yes
Observations	9,080	9,080	9,080	9,080	9,080	9,080
*R* ^2^	0.472	0.489	0.468	0.481	0.471	0.486
Non-manufacturing Sector						
Robot	−0.0052*** (−2.742)	−0.0054*** (−2.813)	0.0016*** (2.742)	0.0018** (2.325)	0.0015**(2.38 6)	0.0017** (2.494)
Controls	Yes	Yes	Yes	Yes	Yes	Yes
Fixed effects	Yes	Yes	Yes	Yes	Yes	Yes
Observations	15,976	15,976	15,976	15,976	15,976	15,976
*R* ^2^	0.551	0.536	0.509	0.528	0.531	0.522

*, **, and *** denote statistical significance at the 10, 5, and 1% levels, respectively.

*t*-statistics are reported in parentheses. Standard errors are clustered at the city level. Individual-level controls include gender, educational attainment, marital status, and hukou status. City-level controls include GDP per capita, population density, and measures of industrial structure. Columns (1), (3), and (5) include city, province-by-year, occupation, and industry fixed effects. Columns (2), (4), and (6) additionally include survey-year-by-occupation and survey-year-by-industry fixed effects. Unless otherwise stated, the remainder of the paper follows the specification in Columns (1), (3), and (5).

Table 3 reports the economic magnitude of the estimates. Since the standard deviation of regional robot penetration is 3.589, a one-standard-deviation increase in robot penetration implies a 2.12 percentage-point decline in subjective health change among manufacturing workers, equivalent to 2.61% of the sample mean. The corresponding effects on objective health and mental health are 0.54 percentage points and 0.0047 index points, equivalent to 0.58 and 0.18% of their respective means. For non-manufacturing workers, a one-standard-deviation increase in robot penetration is associated with a 1.87 percentage-point decline in subjective health change, a 0.57 percentage-point increase in objective health, and a 0.0054-point increase in mental health, corresponding to 2.30, 0.61, and 0.21% of the respective outcome means. These results suggest that the effect on subjective health change is economically more meaningful, while the mental health effects, although statistically significant, remain relatively small in magnitude.

**Table 3:** Economic magnitudes of the baseline estimates.

Outcome	Coefficient on Robot	Effect of 1-SD increase in Robot	Outcome mean	Effect relative to mean
Manufacturing sector				
Subjective health change	−0.0059	−0.0212	0.811	−2.61%
Objective health	−0.0015	−0.0054	0.934	−0.58%
Mental health	−0.0013	−0.0047	2.617	−0.18%
Non-manufacturing sector				
Subjective health change	−0.0052	−0.0187	0.811	−2.30%
Objective health	0.0016	0.0057	0.934	0.61%
Mental health	0.0015	0.0054	2.617	0.21%

The standard deviation of regional robot penetration is 3.589. The effect of a one-standard-deviation increase is calculated as the estimated coefficient on Robot multiplied by 3.589. The relative effect is calculated by dividing this one-standard-deviation effect by the mean of the corresponding outcome.

### 4.2 Differential health consequences of industrial robot adoption: the “education effect” and “uneven health consequences”

To further examine distributional differences in the health effects of industrial robot adoption, we follow Frank (2019) and classify occupations into three skill groups based on average occupational wages. Specifically, occupations with relatively low average wages are defined as low-skill, those with middle-range wages as medium-skill, and those with relatively high average wages as high-skill. Compared with the conventional approach of proxying skills solely by educational attainment, this wage-based classification captures both workers’ human capital and the task content of jobs, and is therefore better suited for analyzing task-biased technological change. We then use both education and occupation-based skill groupings to assess how industrial robot adoption affects workers’ health across different segments of the labor market. In our implementation, we define senior officials and managers in government, Party-affiliated organizations, and public/private enterprises, as well as professional and technical personnel, as high-skill occupations. Clerical and related personnel, together with production and transport equipment operators and related workers, are classified as medium-skill occupations. Workers in commerce and services, as well as those engaged in agriculture, forestry, animal husbandry, fishery, and water conservancy-related production, are classified as low-skill occupations.

Table 4 examines whether the health effects of industrial robot adoption vary by educational attainment. Across all three health outcomes—subjective health change, objective health, and mental health—the coefficient on the interaction term Robot × College (where College = 1 if the individual has a college degree or above, and 0 otherwise) is statistically insignificant in both the manufacturing and non-manufacturing samples. These results suggest that, unlike skill-biased technological change, industrial robot adoption does not exhibit a pronounced “education effect.” In particular, relative to workers with lower educational attainment, the health outcomes of college-educated workers do not display a systematically stronger decline as regional robot penetration increases.

**Table 4:** Robot adoption and workers’ health: education.

Variable	Subjective health change		Objective Health		Mental Health	
	(1)	(2)	(3)	(4)	(5)	(6)
	Manufacturing	Non-manuf.	Manufacturing	Non-manuf.	Manufacturing	Non-manuf.
Robot × college	−0.0001 (−0.018)	0.0010 (0.472)	−0.0001 (−0.019)	0.0007 (0.391)	−0.0004 (−0.047)	0.0009 (0.408)
College	0.0732** (2.410)	0.0861*** (4.517)	0.0714** (2.386)	0.0756*** (4.673)	0.0728** (2.514)	0.0648*** (3.561)
Robot	−0.0065*** (−2.978)	−0.0055** (−2.511)	−0.0018** (−2.365)	0.0015** (2.181)	−0.0012** (−2.054)	0.0014** (2.486)
Observations	9,080	15,976	9,080	15,976	9,080	15,976
*R* ^2^	0.496	0.468	0.452	0.556	0.463	0.551

Table 5 examines whether the health effects of industrial robot adoption differ across occupational skill groups. The results show that the coefficient on Robot × Middle (where Middle = 1 for medium-skill occupations and 0 otherwise) is significantly negative in the manufacturing sample but statistically insignificant in the non-manufacturing sample. By contrast, the coefficient on Robot × Low (where Low = 1 for low-skill occupations and 0 otherwise) is significantly positive in both the manufacturing and non-manufacturing samples. Taken together, these findings indicate that the adverse health effects of robot adoption are larger for medium-skill workers, and that this pattern is driven primarily by workers in the manufacturing sector. In comparison, low-skill workers appear to experience relatively smaller health losses. This evidence is consistent with the task-biased nature of industrial robots and suggests a degree of “uneven health consequences,” whereby robot adoption disproportionately harms the health of medium-skill workers relative to both high-skill and low-skill workers. Notably, however, in the non-manufacturing sample, robot adoption does not generate additional health deterioration among medium-skill workers.

**Table 5:** Robot adoption and workers’ health: occupation.

Variable	Subjective health change		Objective Health		Mental Health	
	(1)	(2)	(3)	(4)	(5)	(6)
	Manufacturing	Non-manuf.	Manufacturing	Non-manuf.	Manufacturing	Non-manuf.
Robot × middle	−0.0028** (−2.018)	−0.0017 (−1.131)	−0.0074*** (−3.352)	−0.0002 (−0.297)	−0.0011*** (−3.632)	0.0016 (0.942)
Robot × low	0.0049** (2.211)	0.0108* (1.693)	0.0061** (2.016)	0.0022*** (3.579)	0.0031*** (3.885)	0.0023* (1.779)
Robot	−0.0056** (−2.541)	0.0021 (0.972)	−0.0054*** (−5.897)	−0.0005 (−0.313)	−0.0018** (−2.461)	0.0013 (0.611)
Observations	9,080	15,976	9,080	15,976	9,080	15,976
*R* ^2^	0.503	0.462	0.548	0.429	0.371	0.451

### 4.3 Instrumental variable (IV) estimation

Directly using domestic industrial robot adoption to construct regional robot penetration may give rise to endogeneity concerns. On the one hand, robot adoption within a country reflects not only exogenous, industry-specific technological trends but may also be correlated with local factors that affect labor demand, leading to omitted-variable bias. On the other hand, in regions where workers’ health is relatively poor, firms may face constraints on effective labor supply and heightened employment risks, which could induce greater reliance on capital deepening through robot adoption. Such responses would increase local robot penetration and generate reverse causality. To better identify the causal relationship between workers’ health and industrial robot adoption, we instrument China’s regional robot penetration using industry-level robot adoption in the United States, constructed as described above. We then re-estimate the model using a two-stage least squares (2SLS) approach.

Table 6 reports the instrumental-variable estimates of the effects of industrial robot adoption on workers’ health, as well as the distributional patterns of these effects. In the first stage, the instrument enters with a positive and statistically significant coefficient, indicating a strong association between industry-level robot intensity in China and that in the United States. Moreover, the first-stage F-statistic is well above the conventional threshold of 10, alleviating concerns about weak instruments. The second-stage 2SLS results show that, for workers in the manufacturing sector, industrial robot adoption has statistically significant adverse effects on all three health outcomes. A 0.74 percentage-point decline in subjective health change, a 0.10 percentage-point rise in the likelihood of chronic disease, and a 0.0011-point reduction in mental health. For workers in the non-manufacturing sector, a one-unit increase in regional robot penetration is associated with a 0.69 percentage-point decrease in subjective health change, but a 0.21 percentage-point increase in objective health and a 0.0018-point increase in mental health, respectively. Regarding distributional effects, the IV estimates do not reveal a pronounced “education effect,” whereas they do point to a pattern consistent with “uneven health consequences,” particularly among manufacturing workers. Overall, the IV results are broadly consistent with the baseline OLS estimates.

**Table 6:** Robot adoption and workers’ health (IV–2SLS).

Variable	Subjective health change		Objective health		Mental health	
	(1)	(2)	(3)	(4)	(5)	(6)
	Manufacturing	Non-manuf.	Manufacturing	Non-manuf.	Manufacturing	Non-manuf.
Panel A. Second-stage (2SLS)						
Robot	−0.0074*** (−3.764)	−0.0069*** (−4.286)	−0.0010** (−2.146)	0.0021*** (2.731)	−0.0011* (−1.874)	0.0018* (1.912)
Panel B. First stage						
Instrument	0.3368*** (10.944)	0.3485*** (14.238)	0.5487*** (46.811)	0.5562*** (27.466)	0.5594*** (46.952)	0.5631*** (27.603)
First-stage F-statistic	119.771	202.72	2191.27	754.382	2204.489	761.926
Panel C. Education interaction						
Robot × college	0.0018 (0.521)	0.002 (0.486)	0.0015 (0.574)	0.0001 (0.031)	−0.0031** (−2.037)	0.004 (0.891)
Panel D. Occupation-skill interactions						
Robot × middle	−0.0039* (−1.821)	−0.0024 (−1.012)	−0.0084*** (−3.334)	0.0005 (0.417)	−0.0011*** (−3.108)	0.0022 (0.603)
Robot × low	0.0078** (2.148)	0.0207** (2.384)	0.0075* (1.913)	0.0028*** (3.781)	0.0026*** (3.417)	0.002 (1.084)
Observations	9,080	15,976	9,080	15,976	9,080	15,976

### 4.4 Validity of the shift-share instrument

#### 4.4.1 Controlling for baseline manufacturing structure interacted with year fixed effects

A first concern is that cities with a larger initial manufacturing base may follow different trajectories over time in ways that are correlated with both robot exposure and workers’ health. To address this, we augment the 2SLS specification by including the city’s baseline manufacturing share interacted with year fixed effects. This flexible control allows cities with different initial degrees of industrialization to follow different time paths, thereby reducing the concern that the shift-share instrument is proxying for persistent city-level industrial structure rather than exogenous automation exposure.

Table 7 shows, after adding this interaction term, the main 2SLS estimates remain stable in sign and broadly similar in magnitude. This suggests that the instrument is not simply capturing differential time trends associated with initial manufacturing intensity.

**Table 7:** Controlling for baseline manufacturing share × year fixed effects.

Variable	Subjective health change		Objective health		Mental health	
	(1)	(2)	(3)	(4)	(5)	(6)
	Manufacturing	Non-manuf.	Manufacturing	Non-manuf.	Manufacturing	Non-manuf.
Panel A. Second-stage (2SLS)						
Robot	−0.0072*** (−3.601)	−0.0066*** (−3.994)	−0.0009* (−1.864)	0.0018** (2.211)	−0.0010* (−1.756)	0.0016* (1.741)
Panel B. First stage						
Instrument	0.3015*** (9.882)	0.3194*** (11.734)	0.5126*** (41.882)	0.5284*** (24.261)	0.5191*** (42.109)	0.5332*** (24.482)
First-stage F-statistic	97.654	137.681	1754.223	588.41	1773.561	596.204

#### 4.4.2 Pre-treatment balance tests at the city level

A second concern is that the instrument may predict pre-treatment local conditions that are themselves related to later health outcomes. To evaluate this possibility, we conduct a set of pre-treatment balance tests. Because CFPS begins in 2010, we cannot observe a long pre-treatment panel of individual health outcomes prior to the onset of large-scale robot diffusion in China. We therefore assess the validity of the shift-share IV using pre-treatment city-level characteristics. Specifically, we examine whether the IV predicts baseline industrial structure, medical resources, pollution exposure, income, and demographic conditions in 2005. If the IV were primarily capturing persistent local conditions rather than exogenous variation in automation exposure, it would be expected to predict these pre-treatment city characteristics.

**Figure 3:**
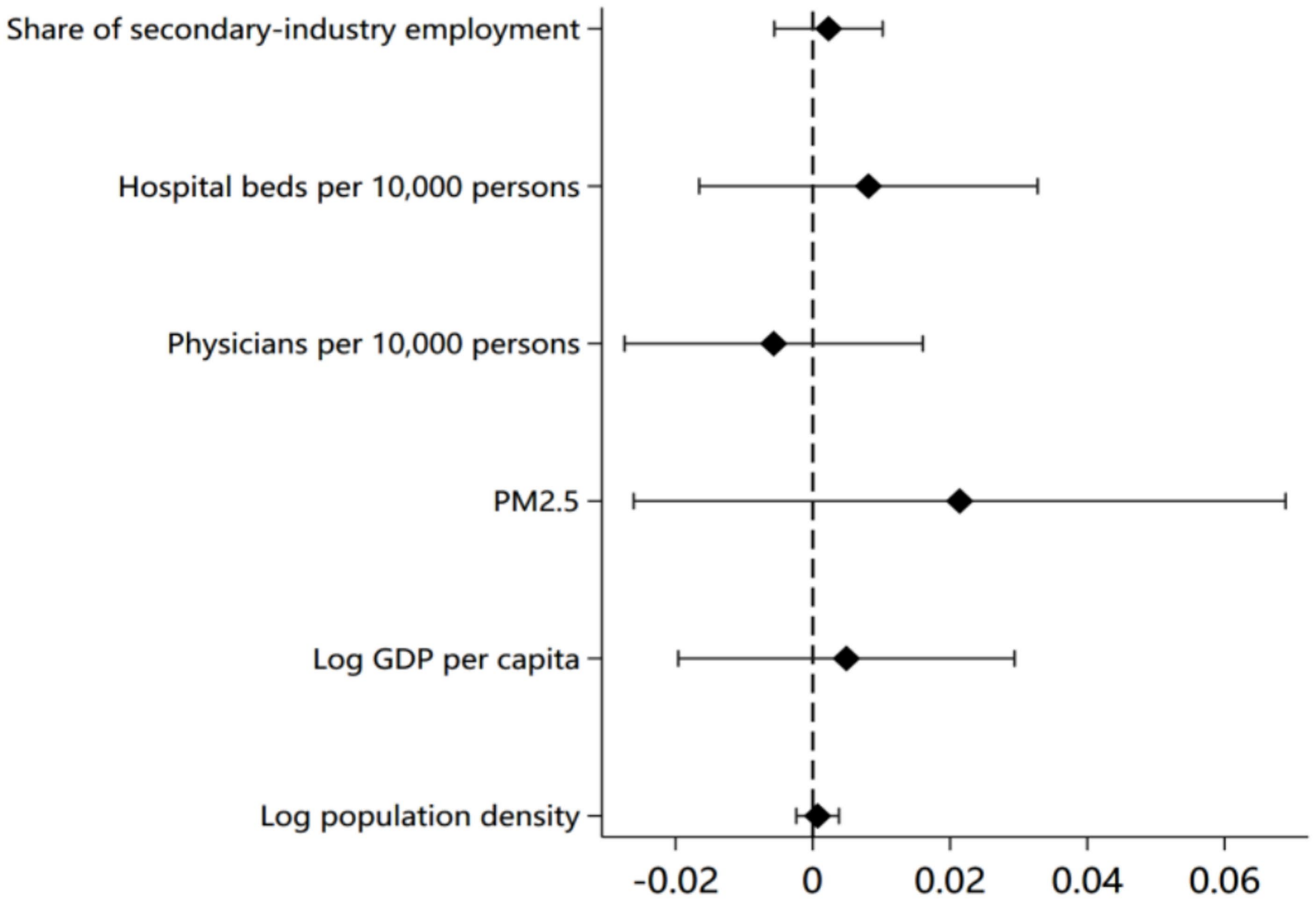
Pre-treatment balance tests at the city level.

The results, reported in Figure 3, show that the IV is not systematically correlated with these pre-treatment city characteristics. This evidence supports the exclusion restriction by suggesting that the instrument is unlikely to be driven by pre-existing local conditions that may later affect worker health independently of robot adoption.

#### 4.4.3 Alternative IV based on multiple advanced economies

To reduce reliance on U.S. robot growth alone, we construct an alternative shift-share instrument using industry-level robot growth in three European economies—Italy, Denmark, and the Netherlands. Specifically, we calculate the average industry-level growth in robot adoption across these three countries and use it to build an alternative measure of external automation shocks. The data for Italy, Denmark, and the Netherlands are drawn from the EU KLEMS database. This alternative instrument captures broader international automation trends and helps address the concern that the baseline IV may be driven by shocks specific to the United States.

Table 8 shows that the 2SLS estimates are qualitatively similar to those obtained using the U.S.-based instrument. In particular, robot adoption remains negatively associated with all three health outcomes in manufacturing, while the estimates for non-manufacturing continue to show differentiated patterns across health dimensions. Although the coefficients are somewhat smaller in magnitude, the overall pattern remains broadly consistent with the baseline IV results, suggesting that the main findings are not uniquely dependent on the U.S. component of the national shock.

**Table 8:** Alternative IV based on multiple advanced economies.

Variable	Subjective health change		Objective health		Mental health	
	(1)	(2)	(3)	(4)	(5)	(6)
	Manufacturing	Non-manuf.	Manufacturing	Non-manuf.	Manufacturing	Non-manuf.
Panel A. Second-stage (2SLS)						
Robot	−0.0071*** (−3.548)	−0.0064*** (−4.116)	−0.0009** (−2.041)	0.0020** (2.364)	−0.0011* (−1.842)	0.0018* (1.781)
Panel B. First stage						
Instrument	0.2984*** (9.874)	0.3147*** (12.661)	0.5218*** (44.283)	0.5341*** (25.714)	0.5269*** (44.596)	0.5388*** (25.988)
First-stage F-statistic	97.496	160.301	1960.987	661.209	1988.803	675.376

### 4.5 Robustness tests

#### 4.5.1 Occupational major-group classification

**Figure 4:**
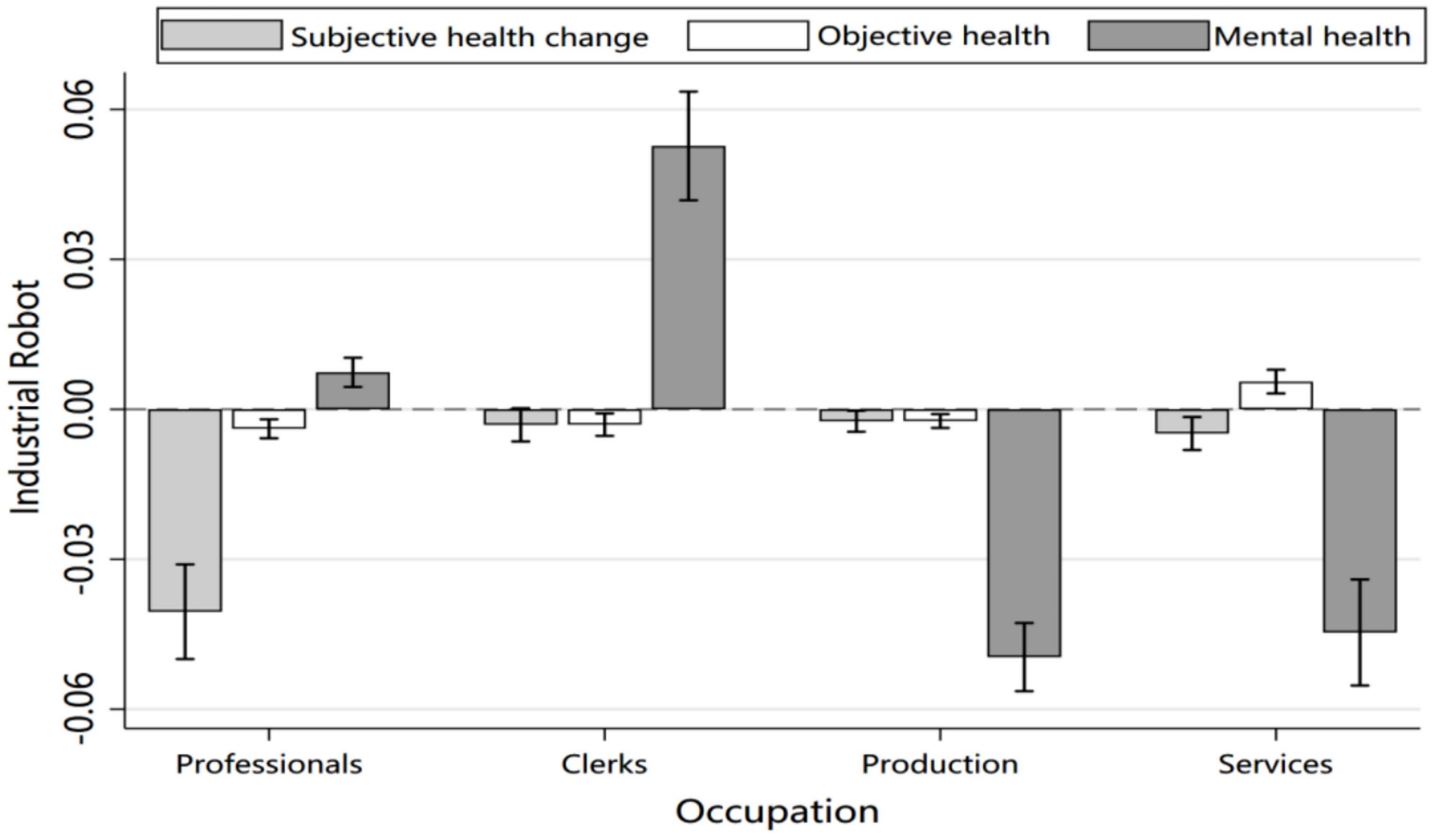
Industrial robot and workers’ health by occupation.

In the baseline analysis of occupation-based skill groups, we classify occupations into high-, medium-, and low-skill categories. However, even within the same skill tier, jobs can differ substantially in task content. To further assess whether the health effects of industrial robot adoption vary with occupational tasks, we exploit China’s official occupational classification and conduct a robustness exercise based on broad occupational groups. Following the occupational major-group taxonomy in the National Occupational Classification of the People’s Republic of China, occupations are divided into eight major categories. Because several groups (e.g., senior officials, agricultural production workers, military personnel, and residual categories) have relatively small sample sizes and are less tightly connected to industrial robot exposure, we focus on four major groups: (i) professional and technical personnel; (ii) clerical and related workers; (iii) production, transport, and construction workers; and (iv) commercial and service workers. We then re-estimate the baseline specification separately for each group to examine whether robot adoption affects health differently across occupations. Figure 4 shows that the adverse health effects of robot adoption are concentrated among production, transport, and construction workers—a group that largely overlaps with medium-skill, routine-task occupations. For this group, industrial robot adoption is associated with significant deteriorations in subjective health change, objective health, and mental health. In contrast, the signs and statistical significance differ across other occupational groups. For professional and technical personnel, robot adoption is significantly negatively associated with subjective health change and objective health, while the association with mental health is weaker and differs in sign. For clerical and related workers, robot adoption is positively associated with mental health, but remains negatively associated with self-assessed and objective health. For commercial and service workers, robot adoption is positively associated with subjective health change and mental health, whereas the pattern for objective health is not aligned with the other two measures.

#### 4.5.2 Controlling for other technological Progress

To rule out confounding influences from technological progress correlated with robot adoption, we further augment the baseline specification by controlling for city-level information technology (IT) development. We operationalize IT development in two ways. First, we use the logarithm of the number of broadband Internet subscribers in a city as a proxy for local Internet development (lninter). Second, we construct a regional measure of computer penetration (Computer), defined as in Equation 4:


$$\text{Compute}r_{\mathrm{ct}}=\sum_{s}\frac{\text{emplo}y_{\mathrm{sc}t_{0}}}{\text{emplo}y_{ct_{0}}}\frac{\text{Compute}r_{\mathrm{st}}}{L_{\mathrm{sc}t_{0}}}$$ (4)

where computers_st_ denotes computer use in China’s industry *s* in year *t*, drawn from the China *Economic Census Yearbook*. All remaining components—base-year employment weights, industry aggregation, and the city-level shift–share structure—are defined analogously to the regional industrial robot penetration measure described above. Relative to city-level proxies such as Internet development, regional computer penetration more directly captures industry-level information technology progress on the production side (i.e., a technology shock affecting firms’ production processes). Controlling for this measure therefore allows us to more cleanly isolate the health effects attributable to robot adoption from broader IT-related technological change. As shown in Table 9, after controlling for either city-level Internet development or regional computer penetration, the estimated coefficient on regional industrial robot penetration remains consistent with our expectations across subjective health change, objective health, and mental health, indicating that the paper’s main conclusions are broadly robust to accounting for concurrent IT progress.

**Table 9:** Controlling for other technological progress.

Variable	Subjective health change		Objective health		Mental health	
	(1)	(2)	(3)	(4)	(5)	(6)
	Manufacturing	Non-manuf.	Manufacturing	Non-manuf.	Manufacturing	Non-manuf.
Panel A. Internet development						
Robot	−0.0071*** (−3.684)	−0.0009 (−0.118)	−0.0031*** (−4.612)	0.0025* (1.721)	−0.0019** (−2.274)	0.0019*** (3.918)
lninter	−0.0081	−0.0004	−0.0247	0.0072	−0.0228	0.0069
	(−0.774)	(−0.046)	(−0.731)	(0.274)	(−0.706)	(0.171)
Panel B. Computer penetration						
Robot	−0.0054* (−1.694)	−0.0027*** (−4.967)	−0.0020* (−1.746)	0.0027* −1.756	−0.0018* (−1.744)	0.0014* −1.689
Computer	−0.0312 (−0.421)	−0.0334 (−0.472)	0.1028* (1.642)	0.1084* (1.742)	0.2517*** (2.804)	0.0062*** (2.931)
Observations	9,080	15,976	9,080	15,976	9,080	15,976

#### 4.5.3 Alternative outcome measures

To further assess robustness, we replace the core health outcomes using data from the China Health and Nutrition Survey (CHNS). Following Li and Wu (31) and Ng et al. (32), we construct a binary indicator of physical health. Specifically, (Physical = 1) if the respondent reports no illness or injury during the past 4 weeks, and (Physical = 0) otherwise. In addition, drawing on Miao and Wu (33), we proxy mental health using the CHNS question “How do you feel about your life?,” denoted as Psycholo. Responses are coded on a five-point scale from 1 (“very bad”) to 5 (“very good”), with higher values indicating better psychological well-being. As shown in Table 10, regressions using these alternative health measures yield results that are qualitatively consistent with our main findings.

**Table 10:** Alternative outcome measures.

Variable	Physical		Psycholo	
	(1)	(2)	(3)	(4)
	Manufacturing	Non-manuf.	Manufacturing	Non-manuf.
Robot	−0.0134*** (−3.972)	0.0119*** (−5.214)	−0.0019*** (−2.648)	0.0035* (−1.781)
Observations	7,686	12,560	7,686	12,560

#### 4.5.4 Other robustness tests

To further examine the robustness of the baseline findings, we conduct several additional tests that are directly related to the measurement and timing of robot exposure. First, we replace the robot penetration measure constructed from industrial robot installations with an alternative measure based on the operational stock of industrial robots (11). Second, because robot adoption is highly concentrated in the automobile and electronics industries, we reconstruct regional robot penetration after excluding these high-robot industries. Third, we use longer lags and cumulative measures of robot exposure to allow for the possibility that workers’ health responds to automation shocks with a delay. Overall, these results in Table 11 support the qualitative robustness of the baseline findings, suggesting that the main conclusions are not driven by a particular robot exposure measure, by a small number of highly robotized industries, or by the use of one-year lagged robot exposure in the baseline specification.

**Table 11:** Other robustness tests.

Variable	Subjective health change		Objective health		Mental health	
	(1) Manufacturing	(2) Non-manuf.	(3) Manufacturing	(4) Non-manuf.	(5) Manufacturing	(6) Non-manuf.
Panel A. Alternative robot exposure measure						
Robot	−0.0073*** (−3.284)	−0.0069*** (−3.114)	−0.0020*** (−2.643)	0.0027*** (2.812)	−0.0016** (−2.114)	0.0025** (2.208)
Panel B. Excluding automobile and electronics						
Robot	−0.0062** (−2.541)	−0.0059*** (−2.874)	−0.0017** (−2.146)	0.0022** (2.201)	−0.0013* (−1.874)	0.0021* (1.936)
Panel C. Three-year lagged robot exposure						
Robot	−0.0058** (−2.314)	−0.0056** (−2.281)	−0.0015* (−1.864)	0.0020* (1.978)	−0.0011 (−1.562)	0.0018 (1.511)
Panel D. Three-year moving average robot exposure						
Robot	−0.0075*** (−3.418)	−0.0070*** (−3.087)	−0.0022** (−2.218)	0.0028** (2.364)	−0.0017** (−2.041)	0.0026** (2.117)

## 5 Mechanism analysis and heterogeneity

### 5.1 Mechanism tests

#### 5.1.1 Labor-market adjustment channel in manufacturing

The theoretical discussion above suggests that a key channel through which industrial robot adoption directly affects the health of manufacturing workers operates through labor substitution and the resulting deterioration in workers’ labor-market position. When the displacement effect dominates, greater substitutability of robots for labor may reduce firms’ reliance on labor inputs and increases workers’ exposure to unfavorable employment conditions. In practice, this may be reflected in lower wages, longer working hours, weaker contractual protection, or reduced access to employment-related benefits, thereby adversely affecting workers’ health. Following the rent-sharing literature, we use workers’ wages as a relatively direct labor-market outcome reflecting workers’ ability to share in productivity gains and bargaining outcomes (34, 35). When workers occupy a stronger position in the labor market, they are more likely to benefit from productivity improvements through better pay and improved employment conditions. By contrast, if robot adoption mainly operates through the displacement effect, higher robot penetration may reduce the extent to which workers benefit—in health terms—from higher wages and related labor-market gains. The estimating equation is specified in Equation 5:


$$\begin{array}{c} y_{\mathrm{ict}}=\lambda +\upsilon {\text{Robot}}_{\mathrm{ct}-1}+\xi {\text{Robot}}_{\mathrm{ct}-1}\times \ln{\text{wage}}_{\mathrm{ict}-1}+\zeta \ln{\text{wage}}_{\mathrm{ict}-1}\\ +\phi X_{\mathrm{ict}}+\eta Z_{\mathrm{ct}-1}+\sigma_{c}+\delta_{m}+\phi_{n}+\theta_{\mathrm{pt}}+\varepsilon_{\mathrm{ict}} \end{array}$$ (5)

where lnwage_ict-1_ denotes the logarithm of wage of individual *i* in city c in year *t*–1. All other variables are defined consistently with the baseline specification. The coefficient ξ is the parameter of primary interest. It captures whether the health-related benefits associated with higher wages are attenuated in places with greater robot penetration, which is consistent with a weaker worker position under displacement-dominated labor-market adjustment.

The regression results are reported in Table 12. Across all three health measures—subjective health change, objective health, and mental health—the coefficient on the interaction term Robot × lnwage is significantly negative for manufacturing workers. This suggests that, as regional industrial robot penetration increases, workers derive fewer health-related benefits from higher wages. Further disaggregating by occupation, the negative interaction effect is particularly pronounced for workers in production, transport, and related occupations, which are more susceptible to robot substitution. These findings are consistent with the interpretation that robot adoption may reduce the extent to which manufacturing workers benefit, in terms of health outcomes, from favorable labor-market conditions. Accordingly, the estimated health effects of industrial robot adoption tend to be more adverse for manufacturing workers, particularly those in production and transport-related occupations.

**Table 12:** Labor-market adjustment channel in manufacturing.

Variable	Subjective health change		Objective health		Mental health	
	(1)	(2)	(3)	(4)	(5)	(6)
	Manufacturing	Production and transport	Manufacturing	Production and transport	Manufacturing	Production and transport
Robot	−0.0049 (−1.582)	−0.0053 (−1.544)	−0.0021* (−1.701)	−0.0018** (−2.083)	−0.0026* (−1.643)	−0.0021** (−2.104)
Robot × lnwage	−0.0197* (−1.703)	−0.0209* (−1.726)	−0.0228* (−1.719)	−0.0241** (−2.204)	−0.0234* (−1.734)	−0.0152** (−2.263)
lnwage	0.0216	−0.0238	0.0187	0.0194	0.0199	0.0112
	(1.021)	(−0.472)	(0.814)	(0.146)	(0.836)	(0.158)
Observations	9,080	7,687	9,080	7,687	9,080	7,687

#### 5.1.2 Potential cross-sector spillover channels in non-manufacturing

Given the central role of the service sector in economic growth and employment creation (36, 37), we further examine whether industrial robot adoption is associated with health outcomes among workers in non-manufacturing—particularly services. The estimation results are reported in Table 13. To capture the potential crowding-out channel, we define lnserve_ct_ as the logarithm of the ratio of service-sector employment to manufacturing employment in city *c* in year *t*. This variable reflects the relative growth of service-sector labor compared with manufacturing; higher values indicate faster service-sector labor growth. We test the potential crowding-out effect using the interaction term Robot × lnserve. The results show that for subjective health change, the interaction coefficient is significantly negative both for the broad non-manufacturing sample and for the service-sector subsample. This indicates that when service-sector employment grows faster relative to manufacturing, industrial robot adoption is associated with a stronger crowding-out spillover on subjective health change among non-manufacturing workers.

**Table 13:** Cross-sector spillovers: non-manufacturing workers’ health.

Variable	Subjective health change		Objective health		Mental health	
	(1)	(2)	(3)	(4)	(5)	(6)
	Non-manufacturing	Services	Non-manufacturing	Services	Non-manufacturing	Services
Panel A. Crowding-Out spillover						
Robot	−0.0064*** (−4.118)	−0.0070*** (−4.702)	0.0025** (2.284)	0.0024** (2.012)	0.0024** (2.176)	0.0026** (2.034)
Robot × lnserve	−0.0053*** (−3.114)	−0.0059*** (−2.961)	0.0018* (1.664)	0.0024 (1.442)	0.0015* (1.658)	0.0014 (1.438)
lnserve	0.0147** (2.201)	0.0131*** (2.986)	−0.0026 (−0.034)	0.0118 (0.228)	−0.0014 (−0.043)	0.0132 (0.239)
Panel B. Crowding-In spillover						
Robot	−0.0055*** (−3.971)	−0.0058*** (−4.053)	0.0018 (0.712)	0.0026 (1.291)	0.0009 (0.701)	0.0015 (1.246)
Robot × high	0.0098 (0.968)	0.0137 (0.924)	0.0034* (1.701)	0.0149* (1.744)	0.0043* (1.722)	0.0161* (1.756)
Observations	15,976	13,464	15,976	13,464	15,976	13,464

One possible explanation is that industrial robot adoption enables firms to substitute machines for workers in routine and codifiable manufacturing tasks, thereby reducing labor demand in the corresponding jobs and pushing some displaced workers to reallocate into non-manufacturing employment. When such inflows are sufficiently large to generate a substantial labor-supply shock in non-manufacturing, robot adoption may intensify employment competition and work-related stress, constraining improvements in non-manufacturing workers’ health. Notably, the results for objective health and mental health differ. For the overall non-manufacturing sample, the coefficient on Robot×lnserve is positive at the 10% significance level. However, once the sample is restricted to the service sector, the interaction term becomes statistically insignificant. This pattern suggests no clear evidence of a crowding-out spillover from industrial robot adoption on objective health or mental health through within–non-manufacturing labor reallocation.

Prior research suggests that, due to human-capital externalities, high- and low-skill workers are strongly complementary: the agglomeration of high-skill labor can foster knowledge spillovers, accelerate technology diffusion, and improve the provision of local public services (36, 38). Accordingly, industrial robot adoption may enhance the work environment in the service sector through the externalities generated by high-skill agglomeration, thereby improving service-sector workers’ health and contributing to a crowding-in spillover. To test this channel (Panel B of Table 13), we use the interaction term Robot×High, where High denotes the city-level share of high-skill workers measured in 2010. High-skill workers are defined as employed individuals with a junior college degree or above (i.e., associate degree or higher).

The regression results indicate that for objective health and mental health, the coefficient on Robot×High is positive and statistically significant at the 10% level in both the broad non-manufacturing sample and the service-sector subsample. This suggests that the positive cross-sector spillover from industrial robot adoption to the health of non-manufacturing workers—particularly service-sector workers—is stronger in cities with a higher baseline share of high-skill labor. By contrast, for subjective health change, the interaction term Robot×High is statistically insignificant in both samples, providing no evidence that high-skill agglomeration amplifies the effect of robot adoption on subjective health change.

Taken together, the evidence suggests that industrial robot adoption is associated with a negative cross-sector spillover in subjective health change among non-manufacturing workers, particularly those in services, whereas the corresponding positive spillover channel is not statistically supported. In contrast, for objective health and mental health, we find evidence more consistent with positive cross-sector spillovers, while the crowding-out channel appears weak or statistically insignificant. A plausible explanation for this divergence is that the three health measures capture distinct dimensions of health and respond differently to external shocks. Subjective health change reflects a subjective, overall assessment and is more sensitive to expectations about job stability, changes in work intensity, perceived relative status, and risk perceptions. When robot adoption increases employment uncertainty, individuals’ self-assessments may deteriorate more readily, which is consistent with a crowding-out spillover in subjective health change. By comparison, objective health and mental health are more likely to improve through resource- and environment-related channels. In particular, the human-capital externalities associated with high-skill agglomeration—induced or amplified by robot adoption—may more directly translate into observable health improvements and reduced psychological strain, making the results more consistent with a stronger and statistically detectable positive cross-sector spillover. A key limitation is that the estimates for non-manufacturing workers rely on city-level robot exposure. As a result, they may reflect not only cross-sector spillovers from robot adoption in manufacturing, but also other concurrent city-level shocks correlated with robot diffusion. The results should therefore be interpreted as suggestive rather than definitive evidence of cross-sector spillovers.

### 5.2 Heterogeneity analysis

#### 5.2.1 Age structure of workers

We examine heterogeneity by workers’ age by dividing the sample into three groups: 20–35, 35–45, and 45–60 years old. Table 14 reports the regression results on the health effects of industrial robot adoption across these age groups. For manufacturing workers, robot adoption is associated with significant deteriorations in subjective health change, objective health, and mental health for those aged 20–35 and 45–60, while the effects for workers aged 35–45 are not statistically significant. For non-manufacturing workers, robot adoption significantly worsens subjective health change across all age groups; by contrast, its effects on objective health and mental health are not statistically significant for any age group.

**Table 14:** Heterogeneity analysis.

Variable	Age 20–35		Age 35–45		Age 45–60		Male		Female	
	(1)	(2)	(3)	(4)	(5)	(6)	(7)	(8)	(9)	(10)
	Manufacturing	Non-manuf.	Manufacturing	Non-manuf.	Manufacturing	Non-manuf.	Manufacturing	Non-manuf.	Manufacturing	Non-manuf.
Subjective health change										
Robot	−0.0066** (−2.184)	−0.0064* (−1.702)	−0.0055 (−1.438)	−0.0077*** (−3.512)	−0.0065*** (−4.106)	−0.0079*** (−4.214)	−0.0048 (−1.334)	−0.0003 (−0.198)	−0.0031 (−1.221)	−0.0002 (−0.084)
Objective health										
Robot	−0.0019*** (−2.541)	−0.0018 (−0.015)	−0.0032 (−1.432)	0.0021 (1.118)	−0.0025* (−1.691)	0.0021 (0.451)	−0.0045*** (−7.762)	−0.0038*** (−4.382)	0.0032*** (7.684)	0.0025*** (4.212)
Mental health										
Robot	−0.0025*** (−2.514)	−0.0019 (−0.028)	−0.0039 (−1.248)	0.0034 (1.214)	−0.018*** (−2.714)	0.0027 (0.462)	−0.0041*** (−7.708)	−0.0032*** (−4.311)	0.0039*** (7.826)	0.0029*** (4.276)
Observations	1,243	3,436	3,877	5,383	4,012	6,163	5,482	3,905	2,991	3,598

These findings suggest that the substitution effects of industrial robot adoption are more pronounced for younger (20–35) and older (45–60) manufacturing workers. Younger workers, who are at an early stage of their careers and possess limited job experience, are more likely to be employed in entry-level, routine, and codifiable tasks that are readily automated. Older workers, although more experienced, often specialize in experiential and routine tasks that are also susceptible to automation; moreover, they may face greater challenges in adapting to new technologies. Consequently, industrial robot adoption tends to exert a significantly negative impact on the health of older workers as well.

#### 5.2.2 Gender composition of workers

The labor-demand and health effects of industrial robot adoption may be asymmetric across men and women. On the one hand, industrial robots are more substitutive for intensive physical tasks than for cognitive work. Because men are more likely than women to be employed in physically demanding jobs, robot adoption may generate larger negative shocks to male labor demand and health. On the other hand, while robots displace certain tasks, they can also create new jobs and reshape task content, potentially offering workers new opportunities to improve health. If the complementarities between workers’ skill endowments and robots differ systematically by gender, robot adoption may therefore have gender-differentiated health effects.

Table 14 reports the estimates by gender. For subjective health change, the coefficient on the interaction term Robot is statistically insignificant in both the manufacturing and non-manufacturing samples. In contrast, for objective health and mental health, the interaction term is significantly negative for male. These results imply that robot adoption does not produce a clear gender gap in subjective health change effects, but it is associated with larger adverse impacts on men’s objective health and mental health outcomes.

## 6 Hukou system, robot adoption and the health of agricultural Hukou workers

The preceding analysis focuses on labor-market mechanisms linking robot adoption to workers’ health. However, the magnitude of these health consequences also depends on the institutional environment. Prior research shows that the hukou system constrains the spatial mobility of labor, contributing to labor-market segmentation (39–42). Moreover, because access to many public services and social insurance programs is tied to hukou status, agricultural hukou holders often face unequal access relative to local urban residents. This institutional constraint raises the cost of job-related migration and limits welfare gains for rural workers (43). The preceding analysis indicates that industrial robot adoption has a pronounced negative effect on the health of workers engaged in production-related jobs. An important question, therefore, is whether hukou constraints amplify this adverse effect for workers with agricultural hukou.

Using CFPS data, we split the sample into workers with agricultural hukou and those with non-agricultural hukou. We then estimate the health effects of industrial robot adoption separately for production, transport, and related occupations within each hukou group. As reported in Table 15, among agricultural-hukou workers, a one-unit increase in regional robot penetration is associated with statistically significant declines in health across outcomes for production, transport, and related workers. In contrast, the corresponding effects are not statistically significant for non-agricultural-hukou workers.

**Table 15:** Hukou system and robot adoption.

Variable	Agricultural Hukou workers			Non-agricultural Hukou workers		
	Subjective health change	objective health	Mental health	Subjective health change	Objective health	Mental health
	(1)	(2)	(3)	(4)	(5)	(6)
Robot	−0.0058*** (−3.318)	−0.0018*** (−3.102)	−0.0024*** (−3.241)	−0.0054 (−0.224)	−0.0015 (−0.251)	−0.0009 (−0.218)
Robot × medical	0.0011** (2.041)	0.0018*** (2.466)	0.0051* (1.706)	0.0008 (0.221)	0.0016 (1.356)	0.0011 (1.284)
Observations	5,334	5,334	5,334	2,353	2,353	2,353

Under the hukou system, agricultural hukou holders often have less comprehensive social protection. As a result, when facing adverse labor-market shocks, they have fewer effective risk-sharing mechanisms, making their health outcomes more vulnerable. More generally, adequate social protection can also strengthen workers’ bargaining position and their capacity to cope with labor-market risks, thereby mitigating health deterioration induced by negative shocks. We therefore further examine whether social insurance attenuates the adverse health effects of industrial robot adoption on agricultural hukou workers (Table 15). The coefficient of primary interest is the interaction term Robot×Medical, where Medical is an indicator equal to 1 if the worker is covered by medical insurance and 0 otherwise. The regression results show that, for agricultural-hukou workers, the interaction coefficient is positive and statistically significant, indicating that medical insurance helps buffer the negative health impacts associated with industrial robot adoption. One caveat is that the objective health measure may reflect not only underlying health status but also access to healthcare and the likelihood of receiving a diagnosis. The buffering effect of medical insurance should therefore be understood as suggestive evidence, especially for the objective health outcome.

## 7 Conclusions and policy implications

This study uses data from the CFPS and constructs multidimensional measures of health—including subjective health change, objective health, and mental health—to examine how industrial robot adoption affects workers’ health in China. We match individual-level outcomes to city-level industrial robot penetration and employ a Bartik-style instrumental-variable strategy to identify causal effects. The results show that for manufacturing workers, robot adoption has significantly negative effects on all three health dimensions. For non-manufacturing workers, however, the effects differ markedly across health measures: robot adoption deteriorates subjective health change but is associated with improvements in objective health and mental health. We further document meaningful distributional patterns. The adverse health effects are larger for workers in medium-skill occupations, consistent with a “uneven health consequences” pattern. Mechanism tests suggest that the direct effects in manufacturing operate through changes in workers’ bargaining position and the distribution of productivity gains, while the findings for non-manufacturing workers provide suggestive evidence of cross-sector spillovers shaped by the degree of substitutability and complementarity across sectors. Additional analyses reveal heterogeneity by age and gender, and we show that hukou-related constraints amplify the negative health impacts for agricultural hukou workers, whereas medical insurance coverage attenuates these adverse effects. Our findings provide an empirical basis for understanding these health consequences in China and for designing policy responses accordingly. The policy implications are as follows:

First, industrial robot adoption is associated with more adverse effects on manufacturing workers across all three health dimensions—subjective health change, objective health, and mental health. These adverse associations are more pronounced for men than for women. One possible explanation is that women are more likely to be employed in non-manufacturing sectors, where the health consequences of robot adoption appear less adverse and where workers may have greater flexibility to adjust to labor-market shocks. More generally, greater flexibility in reallocating across sectors in response to automation-related employment shocks may help buffer the associated health losses. As industrial robot adoption expands, a more balanced and portable system of health insurance and related social protection may reduce the health-related costs of cross-region and cross-sector mobility, strengthen workers’ resilience, and help protect their health. Policy efforts should therefore place greater emphasis on strengthening health-risk protection for manufacturing workers—especially men—who are more exposed to automation-related disruptions.

Second, unlike skill-biased technological change, robot technology exhibits a more strongly task-biased nature and may therefore generate uneven health consequences. Medium-skill workers appear to face relatively larger adverse estimates, which suggests that the health consequences of robot adoption may be uneven across occupational groups. A more cautious implication is therefore that more targeted health insurance design, together with more effective investment in healthcare capacity and resource allocation, may help alleviate adverse health consequences and reduce the risk of widening health disparities across groups.

Third, for agricultural hukou workers, stringent hukou-related constraints amplify the negative health consequences of robot adoption, while medical insurance coverage helps buffer these adverse impacts. These findings point to the importance of strengthening health-risk protection and social protection for worker groups that are more vulnerable to automation-related disruptions. In particular, improving the coverage, accessibility, and targeting of medical insurance may help provide more effective risk sharing for workers facing elevated employment risk, including rural migrants. Such reforms may help mitigate uneven health consequences associated with industrial robot adoption, especially for more vulnerable worker groups.

## Data Availability

The data analyzed in this study is subject to the following licenses/restrictions: The datasets are subject to provider restrictions. CFPS data require registration through the official platform and may not be publicly redistributed by users. IFR robot data are obtained through licensed reports and are not included in the manuscript or supplementary materials. Accordingly, the authors cannot publicly share the full raw datasets; access is governed by the original data providers’ terms. Requests to access these datasets should be directed to CFPS data requests can be made through the official CFPS data platform (https://cfpsdata.pku.edu.cn). Requests regarding IFR World Robotics industrial robot statistics can be directed to the IFR Statistical Department at statistics@ifr.org or via the official World Robotics website.
